# Supplementary material for: Hybrid Models and Biological Model Reduction with PyDSTool
Source: PLoS Comput Biol. 2012 Aug 9;8(8):e1002628. doi: 10.1371/journal.pcbi.1002628 (PMC3415397; doi:10.1371/journal.pcbi.1002628)
Supplement: Text S4 — Complete source code for the PyDSTool package (version 0.88.120504). Includes API documentation and help files linking to web pages. This file is identical to the current public release on Sourceforge.net. (ZIP) [file pcbi.1002628.s004.zip › PyDSTool/html/PyDSTool.ModelSpec'.ModelSpec-class.html]

xml version="1.0" encoding="ascii"?


PyDSTool.ModelSpec'.ModelSpec


| Home | Trees | Indices | Help | PyDSTool |
| --- | --- | --- | --- | --- |

Package PyDSTool ::
Module ModelSpec' ::
Class ModelSpec

[hide private]

[frames] | no frames]

# Class ModelSpec

source code

```
object --+
         |
        ModelSpec
```

Known Subclasses:
:   - Component
    - , LeafComponent

---

Model specification abstract class. Sub-classes can override
targetLangs, compatibleSubcomponents, compatibleContainers, and
compatibleGens.


|  |  |
| --- | --- |
| Instance Methods | [hide private] |

|  |  |
| --- | --- |
| \_\_call\_\_(self) | source code |

|  |  |
| --- | --- |
| \_\_contains\_\_(self, obj) | source code |

|  |  |
| --- | --- |
| \_\_copy\_\_(self) | source code |

|  |  |
| --- | --- |
| \_\_deepcopy\_\_(self, memo=None, \_nil=`[``]`) | source code |

|  |  |
| --- | --- |
| \_\_delitem\_\_(self, name)  Delete named object (Var, Par, Fun, Input, or sub-component) | source code |

|  |  |
| --- | --- |
| \_\_eq\_\_(self, other, diff=False) | source code |

|  |  |
| --- | --- |
| \_\_getitem\_\_(self, name)  Return object named using the hierarchical naming format. | source code |

|  |  |
| --- | --- |
| \_\_init\_\_(self, name)  Called by all sub-classes - do not override | source code |

|  |  |
| --- | --- |
| \_\_ne\_\_(self, other) | source code |

|  |  |
| --- | --- |
| \_\_repr\_\_(self)  str(x) | source code |

|  |  |
| --- | --- |
| \_\_str\_\_(self)  str(x) | source code |

|  |  |
| --- | --- |
| \_infostr(self, verbose=1) | source code |

|  |  |
| --- | --- |
| \_register(self, obj, depth=0, parent\_obj=None) | source code |

|  |  |
| --- | --- |
| add(self, arg, tosubcomponent=None)  Add object to registry, into a specified sub component if provided. | source code |

|  |  |
| --- | --- |
| addConnxnTarget(self, targ) | source code |

|  |  |
| --- | --- |
| compileFuncSpec(self) | source code |

|  |  |
| --- | --- |
| delConnxnTarget(self, targ) | source code |

|  |  |
| --- | --- |
| difference(self, other)  Print the difference between two ModelSpecs to screen. | source code |

|  |  |
| --- | --- |
| flattenSpec(self, multiDefUnravel=True, globalRefs=None, ignoreInputs=False, force=False)  Flatten structured model specification to dictionary compatible with FuncSpec instantiation. | source code |

|  |  |
| --- | --- |
| info(self, verboselevel=1) | source code |

|  |  |
| --- | --- |
| isComplete(self, globalRefs=None) | source code |

|  |  |
| --- | --- |
| isDefined(self, verbose=False, ignoreInputs=False) | source code |

|  |  |
| --- | --- |
| isEmpty(self) | source code |

|  |  |
| --- | --- |
| remove(self, target)  Remove target component from specification. | source code |

|  |  |
| --- | --- |
| rename(self, newName) | source code |

|  |  |
| --- | --- |
| search(self, name, component\_type\_order=None)  Find Quantity objects containing a component named <name>, of type given by the hierarchical name <comptype1.comptype2. | source code |

|  |  |
| --- | --- |
| validate(self) | source code |

**Inherited from `object`**:
`__delattr__`,
`__getattribute__`,
`__hash__`,
`__new__`,
`__reduce__`,
`__reduce_ex__`,
`__setattr__`


|  |  |
| --- | --- |
| Class Variables | [hide private] |

compatibleContainers = `()`

compatibleGens = `()`

compatibleSubcomponents = `()`

targetLangs = `()`


|  |  |
| --- | --- |
| Properties | [hide private] |

**Inherited from `object`**:
`__class__`


|  |  |
| --- | --- |
| Method Details | [hide private] |

|  |  |
| --- | --- |
| \_\_init\_\_(self, name)  *(Constructor)* | source code |

Called by all sub-classes - do not override

Overrides: object.\_\_init\_\_

|  |  |
| --- | --- |
| \_\_repr\_\_(self)  *(Representation operator)* | source code |

str(x)

Overrides: object.\_\_repr\_\_: *(inherited documentation)*

|  |  |
| --- | --- |
| \_\_str\_\_(self)  *(Informal representation operator)* | source code |

str(x)

Overrides: object.\_\_str\_\_: *(inherited documentation)*

|  |  |
| --- | --- |
| flattenSpec(self, multiDefUnravel=True, globalRefs=None, ignoreInputs=False, force=False) | source code |

Flatten structured model specification to dictionary compatible with
FuncSpec instantiation.

Use globalRefs option to declare global variables used in the
definitions that are not defined in them (e.g. time).

Default for multiple quantity definitions is to unravel them. Use
multiDefUnravel=False to override this.

Use force option to rebuild flat spec if an existing one is out of
date.

|  |  |
| --- | --- |
| remove(self, target) | source code |

Remove target component from specification.

Use global hierarchical names for components if specifying a
string.

|  |  |
| --- | --- |
| search(self, name, component\_type\_order=None) | source code |

Find Quantity objects containing a component named <name>, of
type given by the hierarchical name <comptype1.comptype2. ...
.name>, where component\_type\_order = [<comptype1>,
<comptype2>, ... ], and <comptypeN> may be a component type
(as a wildcard) or a specific component name.

  


| Home | Trees | Indices | Help | PyDSTool |
| --- | --- | --- | --- | --- |

|  |  |
| --- | --- |
| Generated by Epydoc 3.0.1 on Fri May 4 15:24:07 2012 | http://epydoc.sourceforge.net |
